# Synthesis of Mesoporous α-Fe_2_O_3_ Nanoparticles by Non-ionic Soft Template and Their Applications to Heavy Oil Upgrading

**DOI:** 10.1038/srep39136

**Published:** 2016-12-14

**Authors:** Chulwoo Park, Jinhwan Jung, Chul Wee Lee, Joungmo Cho

**Affiliations:** 1 Research Center for Convergent Chemical Process, Korea Research Institute of Chemical Technology, Daejeon 34114, Republic of Korea; 2 Kyoungbook National University, Daegu 41566, Republic of Korea; 3 Department of Green Chemistry & Environmental Biotechnology, University of Science and Technology (UST), Daejeon 34113, Republic of Korea

## Abstract

This paper reports the synthetic route of 3-D network shape α-Fe_2_O_3_ from aqueous solutions of iron precursor using a non-ionic polymeric soft-template, Pluronic P123. During the synthesis of α-Fe_2_O_3_, particle sizes, crystal phases and morphologies were significantly influenced by pH, concentrations of precursor and template. The unique shape of worm-like hematite was obtained only when a starting solution was prepared by a weakly basic pH condition and a very specific composition of constituents. The synthesized nanocrystal at this condition had a narrow pore size distribution and high surface area compared to the bulk α-Fe_2_O_3_ or the one synthesized from lower pH conditions. The hydrocracking performance was tested over the synthesized iron oxide catalysts with different morphologies. The worm-like shape of iron oxide showed a superior performance, including overall yield of liquid fuel product and coke formation, over the hydrocracking of heavy petroleum oil.

Iron oxide nanostructures are very important classes of materials used in a variety of fields including catalytic^1^,^2^,^3^,^4^, environmental^5^, medical^6^, and battery^7^ applications. In an industrial aspect, major favorable advantages are that the materials are prepared in a non-toxic and inexpensive manner, but have diverse crystal structures possessing unique properties on their own. The functionality of the materials, such as catalytic activity and magnetic behavior, can be significantly improved when their crystal sizes are confined to the nano-scale and morphologies are controlled to have a high surface area. Hence, many synthetic routes have been suggested to derive ordered and non-ordered nanocrystal structures of iron oxide. Technological advances in nano-material science based on templates have envisioned more elaborate and reliable synthetic routes of mesoporous structure in such materials.

The synthetic methods of the mesoporous metal oxides based on the template used are largely classified into two major routes, i.e. hard- and soft-template methods^8^. In hard template methods^3^,^9^,^10^,^11^,^12^,^13^,^14^, pre-patterned rigid materials are typically used as a host template. In this synthetic route, the hard template plays a role of structural materials restricting crystalline growth to the void space, leading to subsequent replica production. A synthetic route via soft template employs the self-assembly induced by amphiphilic block copolymers or surfactants^2^,^15^,^16^,^17^,^18^. Typically the synthetic routes are carried out in a relatively facile and simple way because it doesn’t require any prior fabrication steps of template, which must be prepared by using another template. However, the method involves complex sol-gel chemistries associating with interactions between surfactants and chemical species comprising the system. In general, synthetic pathways are significantly different, especially with regard to non-silica mesoporous materials, and thus the synthetic method resorting one specific case cannot be widely applicable albeit numerous efforts have been made to synthesize a variety of mesoporous materials with several different morphologies^19^. The synthesis of mesoporous metal oxide using soft-template involves hydrolysis-condensation of metal precursors while the templating agent induces the coordination not only between template molecules but also between template and metal precursor molecules. The coordination of such molecules is directed by non-covalent bonds such as hydrogen bonds or electrostatic bonds. Combining with such complex physicochemical phenomena, formation of reliable self-assembly building structures, susceptibility of solution pH and ionic constituents to metal oxide crystallization, stability of micelle structure against the growth direction of metal oxide, morphologically even crystal-crystal phase transition during thermal treatment, and complete removal of template by post-treatment are very critical and challenging problems in these methods^8^.

Nevertheless, there have been continuous efforts to synthesize iron oxide nanostructure possessing large surface area by deriving various novel morphologies such as hollow tube^20^, rod^21^, sphere^22^, flower^23^, and flute^24^. In most synthetic methods of mesoporous iron oxide, hard templates are favored mainly due to the abrupt gelation characteristics, difficulties in the direct derivation into homogeneous crystal phase and collapse of mesoporous structures during the transitions from one crystal phase to the other desired phase^16^. However, the synthesis of few metal oxides in this method are limited by reactivity with precursors and instability against strong acids or strong bases (e.g. Silica templates can be bleached by HF or NaOH)^11^. Recently, evaporation-induced self-assembly (EISA) strategies^15^,^16^ have been applied to prepare ordered patterns of mesoporous iron oxide films using soft-template. In this synthetic route, silicon or glass is typically used as a substrate. Thermally durable templates are also necessary because the process requires very rigorous temperature upraising to evolve crystal phase transition and development of mesopores wherein organic solvents evaporate. The synthetic routes employing colloidal micellar assembly is much more challenging because it requires controllable rate of sol-gel process and the crystal growth along the micellar surface, which could be initially induced by weak electrostatic interaction between precursor and surface of self-assembly. The restrained hydrolysis and condensation, where organic solvent and iron alkoxide precursors are employed, allows the synthesis of mesoporous non-silica metals oxides, but limited to few transitional metal oxide films^25^. However, the synthesis of mesoporous iron oxide in such systems has not been actively exploited because crystallization from iron precursors generally exhibits a strong gel tendency eventually leading to a bulk or an inconsistent structure, especially when aqueous solution of metal precursors and soft templates (either ionic or non-ionic) are adopted simply.

To our best knowledge, the synthesis of worm-like mesoporous iron oxide using a non-ionic soft template in an aqueous solution has not been reported yet. Herein, we report the α-Fe_2_O_3_ (hematite) nanocrystals having 3-dimentional network with a narrow pore size distribution can be synthesized by controlling acid-base conditions and non-ionic soft template, Pluronic P123. Iron oxide can take diverse crystal phases^26^. Among them, hematite takes a corundum-like structure of crystalline iron oxide that is very stable at an ambient condition and most widely used for several applications including catalysts^1^,^2^,^3^.

The hydrocracking is a major upgrading process to convert heavy hydrocarbon feedstock into light fuel oils by adding hydrogenation catalysts. As worldwide crudes become dirtier and heavier, the slurry-phase hydrocracking has attracted a growing attention from oil refinery industries and related societies because it has many advantages, including feedstock flexibility, high yield of valuable distillate, low coke formation, and consequent process stability, over the other hydrocracking technologies^27^. One major feature of slurry-phase hydrocracking differ from others is that the catalysts are supplied in a form of well-dispersed mixture with heavy hydrocarbon feedstock so that the catalytic reaction can proceed promptly due to their initial close contact. The dispersed catalysts employed in current commercial technologies are largely classified into two types; one is organometallic precursors containing molybdenum and the other is heterogeneous iron oxide catalysts finely pulverized into submicron. Iron oxide catalysts are known to have a lower activity than molybdenum-based catalysts but it has a high potential to reduce the use of expensive metals and to operate the hydrocracking process without losing the overall liquid fuel product yield if the reaction conditions are adjusted. In the catalytic applications of heavy oil hydrocracking, nanosized iron oxide has been employed as an additive (in the commercial technology of KBR’s VCC)^28^,^29^, a supporter^4^, or an active metal component^30^,^31^ for the hydrogenation although their functional roles are not explicitly discriminated.

The mesoporous structure of hematite is expected to have improved hydrogenation ability, especially when their pore sizes are uniformly controlled to have no diffusion limitation of reactant molecules. In this study, the synthesized mesoporous iron oxides were applied to demonstrate catalytic activities in slurry-phase hydrocracking of petroleum residue in which selective conversion of targeting heavy molecules including asphaltenes (their molecular sizes are roughly between 10 and 20 nm^29^) into light hydrocarbon fuels is desired.

## Results and Discussion

**Figure 1:**
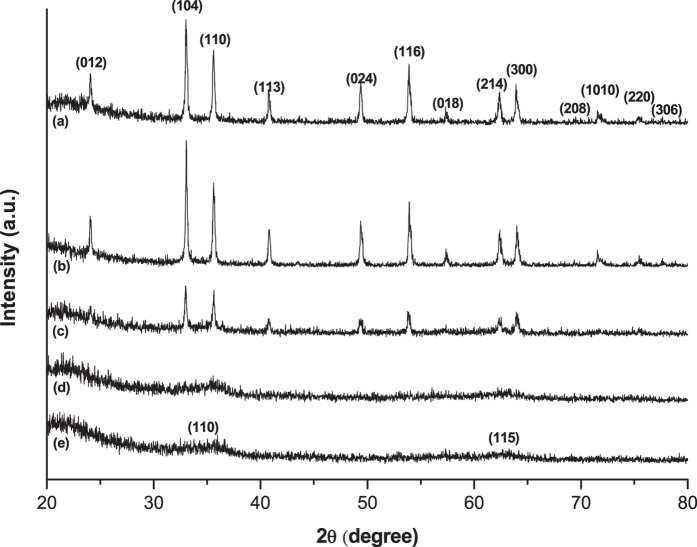
XRD patterns of as-synthesized iron oxides by starting solution at pH (**a**) 5, (**b**) 6, (**c**) 7, (**d**) 8, and (**e**) 9.

Figure 1 shows the X-ray diffraction (XRD) patterns for as-synthesized iron oxide samples using the method described in the experimental section. Although homogeneous iron oxide crystal phases were not obtained, there was an obvious boundary for the transition of one major crystal structure to the other. As-synthesized iron oxide obtained from starting solution of lower pH than 7 displays characteristic peaks indicating mainly hematite (JCPDS 01-080-5405) while the samples obtained from the solution of higher pH exhibit much broader and smaller characteristic peaks near at 2θ = 34.4° and 62.8°. Those are assigned to the (110) and (115) planes of ferrihydrite (JCPDS 29-0712) which can be synthesized by ferric chloride and pH adjustment by NaOH solution as well^32^ During the ageing step, Fe(III) ions in equilibrium with the solution can undergo the following pathways, as proposed by Mao *et al*. in their TG-DSC observation of iron oxide crystal phase transformation^33^:


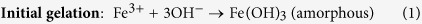



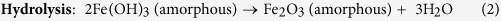



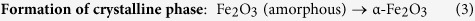


The rate of hematite formation is expedited by an acidity of solution under the thermal treatment, while a basic condition or an insufficient aging time for the acidic solution produces an amorphous or a partially amorphous iron oxide phase oxide phases without further transformation to hematite. The synthesis starting at a very high pH (beyond 9) solution resulted in the crystal structure of goethite(α-FeOOH; JCPDS 81-0463), as XRD patterns for those cases are presented in Fig. S2. The results explain that the control of initial pH in solution significantly affects the final crystal structure even though pH can vary to a small degree during the ripening step.

**Figure 2:**
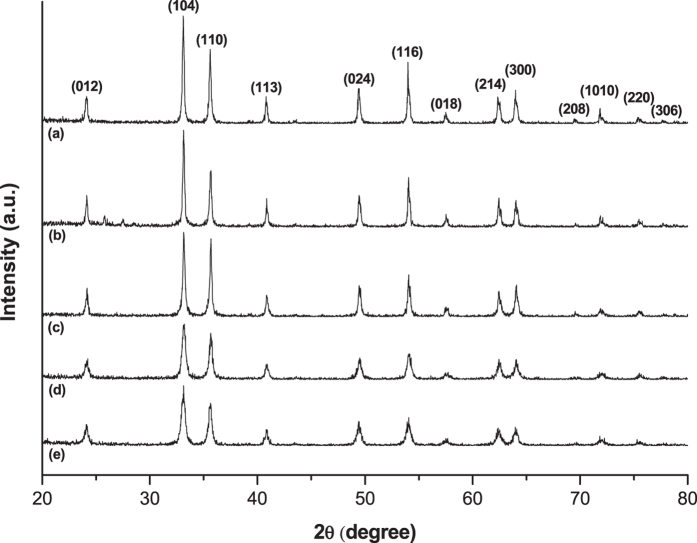
XRD patterns of calcined iron oxides prepared by starting solutions at pH (**a**) 5, (**b**) 6, (**c**) 7, (**d**) 8, and (**e**) 9.

Figure 2 represents XRD patterns for the samples obtained after the calcination. All identified peaks of XRD patterns belong to the characteristic peaks of α-Fe_2_O_3_ crystalline structures (JCPDS 33-0664). However, the (110) peak has a higher intensity than (104) peak for iron oxide synthesized at pH between 7 and 9 when compared to the one at pH conditions between 5 and 6. This result indicates that the direction of α-Fe_2_O_3_ crystalline structure did not conform to the direction of the crystal growth, i.e. [001], in a bulk synthesis of α-Fe_2_O_3_^34^. Comparing two major peaks at between 2θ = 30° and 40°, it is notable that the more broad peaks are observed for the structures derived from the higher pH. The result well coincides with the behavior based on the Scherrer equation which could be more clearly proven with the spherical hematite nanocrystals even for small variation of crystal sizes^22^.

**Figure 3:**
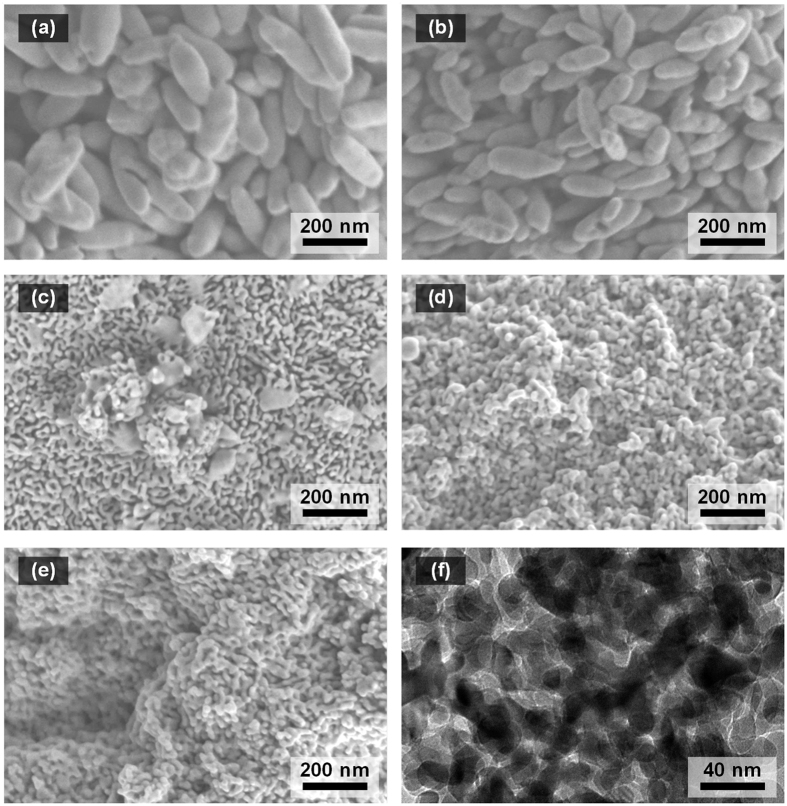
SEM images of calcined iron oxide nanoparticles prepared by starting solutions at pH (**a**) 5, (**b**) 6, (**c**) 7, (**d**) 8, and (**e**) 9. (**f**) is a TEM image for the same sample with (**e**).

The morphology and size for calcined iron oxide nanoparticles prepared within different pH were further investigated by scanning electron microscopy (SEM) and transmission electron microscopy (TEM). Corresponding images are shown in Fig. 3. The synthesis of iron oxide prepared from an acidic condition of pH 5 resulted in an oval-like morphology that has a relatively wide spectrum of particle sizes similar to some synthetic results by acidic solutions of FeCl_3_ in the presence of self-assembled F88 or F127 among other Pluronics by HCl^35^. Diagonal lengths of the particles are between 130 and 390 nm. With a slight pH increase (pH = 6) of the starting solution, significant change of morphology was not observed, whereas the average particle size seems shift slightly downwards (Fig. 3b). The sample derived from the neutral pH was observed to possess mixed crystal structures having obvious two different morphologies (Fig. 3c).

Those are originated from hematite and ferrihydrite phases in an as-synthesis sample which can be further validated by corresponding XRD patterns in Fig. 1. Further increase of pH in a starting solution brought in clear disappearance of bigger particles and only worm-like porous structures remained unaltered significantly (Fig. 3d,e). The observed ridge sizes are about 20–30 nm and the width of void space are about 15–25 nm for the structures. It is notable that such extraordinary shapes are not attainable with a similar pH adjustment using only sodium hydroxide for starting solution (presented in Fig. S4). The result attributes to that the electrosteric effect of micellar structures, initially induced by P123 in an ionic solution with hydrochloric acid, helps anisotropic aggregation of thermodynamically unstable ferrihydrite to the void space of self-assembled structure without significant deformation during the ageing step. The synthesis of worm-like mesoporous structures was successfully prepared only when initial concentrations for iron precursor and surfactant are in a particular range. Meanwhile, no addition of P123 or one of their concentrations was changed to one fold more or less during the synthetic steps, the worm-like structures were not successfully synthesized instead sphere-like particles were produced as their SEM images are shown in Fig. S5 and Fig. S6.

**Figure 4:**
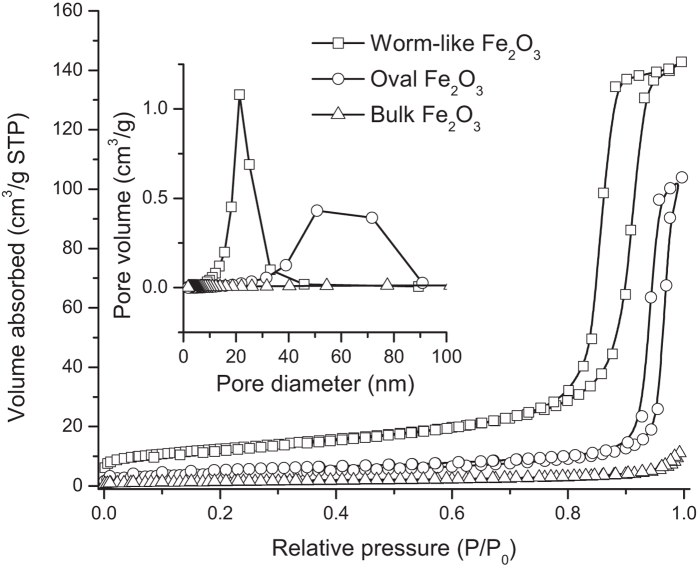
Nitrogen adsorption-desorption isotherms and BJH pore size distributions for bulk α-Fe_2_O_3_, synthesized mesoporous α-Fe_2_O_3_ having shapes of worm-like (started with pH 9 solution) and oval (started with pH 5 solution).

Figure 4 shows nitrogen adsorption-desorption isotherms of the bulk and calcined hematite. The type IV isotherm pattern is clearly observed for the synthesized hematite while the bulk hematite features a flat isotherm. For the samples prepared at pH 5, however, the hysteresis loop is not well established. This poor hysteresis loop is presumably caused by the presence of inter-particle void space. In contrast to the other cases, the sample started from the solution of pH 9 exhibits a well-defined type IV pattern and the behavior corresponds to those of mesoporous hematite presented in other references^3^,^11^,^36^. The inset of Fig. 4 displays BJH plots indicating pore size distributions estimated from desorption isotherms of each sample. A BJH plot for the hematite prepared at pH 9 is observed to have much narrower pore size distribution compared to bulk hematite or one derived from pH 5. Table 1 summarizes surface area, pore volume and average pore size for each sample. Significant increase of BET surface area and smaller average pore size could be observed when the sample is prepared with basic conditions. The samples synthesized at a range of pH 7 to 9 have a relatively sharp pore size distribution and the average sizes are about 20 nm. The results are well consistent with the SEM images. In our extended analysis of low angle XRD (not included here) or TEM image (Fig. 3(f)), any ordered patterns in structure, for both before and after calcination, were not found, but we can conclude that mesopores are well developed through the most domain of crystal structure prepared at a high pH, especially pH 9.

**Figure 5:**
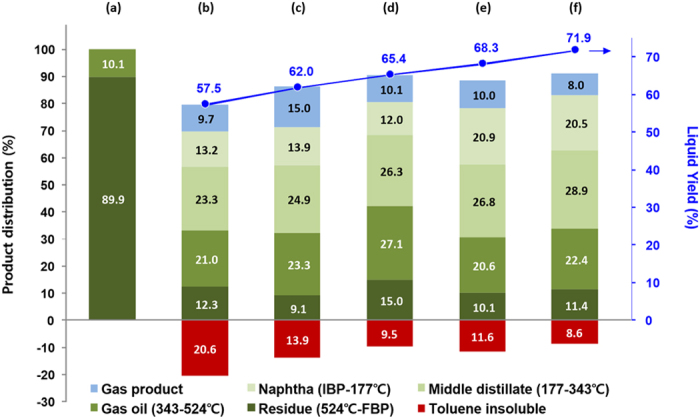
The product distributions after the hydrocracking of (**a**) heavy feedstock when (**b**) no catalysts, (**c**) α-Fe_2_O_3_(pH5), (**d**) α-Fe_2_O_3_(pH9), (**e**) 2% Mo/α-Fe_2_O_3_(pH5), and (**f**) 2% Mo/α-Fe_2_O_3_(pH9) are used. IBP represents the initial boiling temperature at around ambient temperature(25 °C). FBP is the final boiling temperature (between 710 and 740 °C for all samples measured here).

The synthesized mesoporous iron oxide particles and their modified forms were applied to slurry-phase hydrocracking reactions to demonstrate the feasibility of materials as a dispersed catalyst. In the catalytic activity test, a heavy petroleum vacuum residue (VR) was used as a feedstock for the hydrocracking. The detailed properties for the feedstock obtained from chemical analysis are listed in Table S1. It is known that the catalyst amount affects the catalytic performance significantly^4^,^37^. In the current study, small amount of catalysts (0.5 g per 40 g of VR) were chosen to check the distinct difference of catalytic performance in the kinetics limited regime. Figure 5 shows the hydrocracking results of heavy petroleum residue. The products have a wide spectrum of boiling point and their fractions can be classified into the following categories that are conventionally adopted in the petroleum refineries; gas product (typically C1-C5 paraffin molecules) and liquid products which are further divided into naphtha (25–177 °C), middle distillate (177–343 °C), gas oil (343–524 °C) and unconverted residue (>524 °C)^38^. Among hydrocracking products, toluene insoluble, being physically solid polyaromatic particles when separated, is regarded as a coke because most unconverted residual fractions including asphaltenes are soluble in toluene. In the absence of catalysts (Fig. 5(b)), the reaction route to hydrogenation is not promoted by the catalysts and thus only thermal hydrocracking of feedstock undergoes in this condition. In this instance, the hydrocracking of VR induces the large fraction of coke which is facilitated by polycondensation between heavy aromatic compounds and abundant radicals triggered by thermal cracking instead of hydrogen capping. Figure 5(c) and (d) indicates the results of hydrocracking of VR in the presence of synthesized hematite nanoparticles prepared by starting solutions of pH5 and pH9 respectively. As reported^4^,^30^,^31^, iron oxides improved the yield of liquid products and inhibition of coke formation to a significant degree. For the hydrocracking of heavy petroleum residue containing high amount of sulfur (mostly embedded in the heavy molecules), it is known that the iron oxides are mainly converted into the active form of pyrrhotite (Fe_(x−1)_S_x_; JCPDS 29–0723)^39^,^40^. Such major crystal phase transformation was also observed in our cases, however the significant morphology destruction or activity loss was not observed in our extended hydrocracking tests for catalyst recycles. Their detailed descriptions are presented in Fig. S7 through Fig. S10. (According to the convention, we denoted the catalyst as the initial crystalline form of metal oxide, i.e. iron oxide, in this paper.). It is notable that the worm-like iron oxide catalyst having a significant amount of mesopores shows a favorable result in the aspects of liquid product yield and coke formation (less than 10 %). Although a higher fraction of unconverted residue is observed, the higher yield of liquid product (>65%) is observed when worm-like iron oxide is applied. The result implies that enhanced mass transfer of large macromolecules into the internal pore reduces the chance for development of asphaltenes into polyaromatics and the larger surface area of the material may amplify the role of radical scavenger efficiently. The similar methods confining the pore sizes of catalytic supporter were reported by Byambajav and Ohtsuka^31^. They reported the effect of pore sizes of SBA-15 supporter in iron oxide loaded catalysts (Fe/SBA-15) on the hydrocracking of asphaltene. Their result for hydrocracking tests shows a good agreement with the results presented in Fig. 5(b–d) qualitatively although used supporter types and feedstock are quite different from the current study. In their result, the SBA-15 supporter controlled to have a large pore size at between 5–20 nm was found to be more effective in improving the final yield of light liquid product, while the residual conversion regresses steadily for the catalysts having the average pore sizes larger than 12 nm. The slightly lower conversion can be overcome by increase of reaction time in a batch operation or by increase of residence time in a continuous operation of hydrocracking while the amount of irreversible coke formation could not be manipulated simply.

Molybdenum is known to have a higher activity than iron oxide for the hydrogenation^29^. The modified catalysts with synthesized iron oxide nanoparticles were further tested by adding a small amount of molybdenum (Mo 2%). Figure 5(e) and (f) shows the results of hydrocracking over the 2% Mo/α-Fe_2_O_3_ catalysts at the same conditions and their synthesized iron oxide supporters were prepared by starting solutions of pH5 and pH9 respectively. In both cases, the faithful improvement for coke suppression and liquid products yield can be found compared to the cases of original iron oxide particles. In particular, 2%Mo/α-Fe_2_O_3_ (prepared by pH9 solution) catalysts shows an excellent selectivity to valuable liquid products (especially high yield of middle distillates which are most valuable), reduction of less valuable gas products, relatively high residual conversion, and small amount of coke formation. Although we are not quite successful to analyze Mo dispersion over the iron oxides in the analysis of TEM-EDS mapping (mainly because of insufficiently strong detection of molybdenum over the iron oxide specimen), we believe that the better dispersion of limiting amount of Mo over the supporter having a higher surface area may also contribute to improve the hydrocracking performance because there is a higher chance to form single layered MoS_2_ by *in-situ* sulfidation while only a rim side of basal sites in MoS_2_ multilayers possesses an equivalent hydrogen activity. In summary, the results implies that the iron oxide materials having well-developed mesopores and no limitation of asphaltene mobility to internal pores can be a good candidate for the heterogeneous dispersed catalyst or its supporter.

Apart from the parallel comparison between different iron oxide supporters, it is noteworthy to see how the mesoporosities of iron oxide structure affect hydrocracking performance. It is very interesting that unmodified mesoporous iron oxide catalyst (Fig. 5(d)) brings in a comparable liquid product selectivity and less amount of coke formation than Mo supported on the iron oxide particle with fewer amounts of mesopores (Fig. 5(e)). This result reveals that the appropriate pore size control of iron oxide catalysts can be an effective strategy to improve the overall performance of hydrocracking while none of or fewer amount of expensive metals are added.

## Conclusions

In summary, the mesoporous worm-like iron oxide crystal structures were successfully synthesized by pH control of initial solution containing a very common iron nitrate precursor and non-ionic template, P123. Their transformation to homogeneous α-Fe_2_O_3_ phase could be achieved without significant structural deformation when exposed to abrupt oxidation at a high temperature in the presence of air. When started with the solution of [Fe precursor]/[P123] = 1:4 adjusted to pH between 7 and 9, mesoporous α-Fe_2_O_3_ nanoparticles, showing a narrow pore size distribution and 20 nm average pore size, were obtained. The proposed synthetic route is relatively facile and simple without any further uses of expensive reagents or the aid of hard-templates. In the hydrocracking tests over the synthesized iron oxide or Mo loaded iron oxide catalysts, the catalytic supporters with well-developed mesopores were more beneficial in terms of valuable liquid fuel product yield and coke suppression. Although we put only one example of hydrocracking in this contribution, we expect that the suggested fabrication methods for mesoporous hematite will be very useful for other applications, especially for catalysts requiring facilitated mass transport of massive chemical species into the pore, owing to the relatively large open pores in their structures and the potential to scale-up in an efficient and economical way.

## Methods

### Preparation of α-Fe_2_O_3_ nanocrystal structure

The mesoporous hematite structures were prepared in the following procedures. Triblock copolymer Pluronic P123 (EO_20_PO_70_EO_20_, Mn = 5,800) and bulk hematite(Iron(III) oxide, powder, <5 μm) were obtained from Aldrich. All other materials were purchased from Samchun chemical co, Korea unless otherwise specified. All chemicals were used as received and without any further purification. 8 g of Pluronic P123 were added to 60 g of water and stirred for an hour to obtain a completely dissolved solution. The solution was mixed with 240 g of 2 M HCl aqueous solution with continuous stirring for 10 h by magnetic stirrer. Then, 2 g of Fe(NO_3_)_3_·9H_2_O powder was added to the resulting solution. After 5 min stirring, 10 M sodium hydroxide solution was added to the mixture till the solution reaches at pH 5. During the addition of basic solution, the solution temperature was controlled to maintain below 40 °C by adjusting the titration speed. The same procedures were repeated to prepare samples having different pH 6 to 12. After pH adjustment, each sample was transferred to the oven preheated to 100 °C and kept there for 60 h. After this aging step, a large amount of P123 can be recovered because they were entangled massively and floated on the solution. The segregated iron oxide particles were purified by repeating membrane filtration followed by removal of upper solution and replenishing DI water for three times. The final wet solid powders were dried in vacuum oven kept at 60 °C (as-synthesized iron oxide). For a complete removal of soft template residue and transformation to hematite, the calcination was carried out in a box furnace maintained at 500 °C for 5 h under air flow of 100 ml/min.

### The analysis of crystal structure for synthesized iron oxide nanocrystal structure

Crystal structures of synthesized iron oxide were characterized by XRD (Rigaku RINT-2000 vertical goniometer operated at 40 kV/40 mA with Cu Kα radiation). The morphology of calcined α-Fe_2_O_3_ nanocrystals was observed by FE-SEM (FEI Magellan XHR 400) and TEM (TECNAI G^2^ T-20S). From a nitrogen adsorption-desorption isotherm (Micromeritics, ASAP 2420 system) for each sample, the Brunauer-Emmett-Teller (BET) surface areas, the pore diameters by applying Barrett-Joyner-Halenda (BJH) model, and the total pore volumes were determined.

### The modification of iron oxide nanocrystals

To explore potential applications of the materials to slurry phase hydrocracking, synthesized and modified α-Fe_2_O_3_ nanocrystals were prepared for dispersed catalysts. The modifications of α-Fe_2_O_3_ nanocrystals were performed based on the incipient wetness impregnation methods. For instance, 0.076 g ammonium heptamolybdate tetrahydrate (Assay >99.0%, Showa Chemical) was dissolved in pre-calculated amount of D.I. water and then the solution was slowly titrated to 2.062 g of synthesized iron oxide powders with an exposure to sonication (60 Hz) and continuous mixing for 1 h at the temperature of 40 °C. Then the resulting products were dried in an air oven at 120 °C for 12 h and ground with a pestle and a mortar. The dried powder samples were placed to an air furnace, heated to 450 °C by a heating rate of 100 °C/min under the constant air flow of 100 ml/min and held for 1 h more.

### The hydrocracking of heavy oils

The hydrocracking experiments were carried out using an autoclave reactor (vessel volume: 250 ml, model: Parr 4566). 40 g of vacuum residue (from Hyundai Oilbank Co.) and 0.5 g of catalysts (for catalytic reactions only) were added to the reactor and the reactor temperature was raised to 80 °C. The hydrogen gas (99.999%) purging steps were repeated more than 3 times and the final hydrogen fill-up was made to keep 80 bars in gauge at an equilibrium temperature of 80 °C. After halting H_2_ flow, the reactor was heated up to the reaction temperature 430 °C with a nominal heating rate of 15  °C/min and the hydrocracking reactions were performed for 2 h. After the reaction, the reactor was suddenly chilled with cooling water till it reaches an ambient temperature. Gas sample was collected with 20 L Tedlar bag, and liquid and precipitate samples were transferred into the glass vials for further analysis followed by weighing with a high-precision digital balance.

### The analysis of feedstock and hydrocracking products

Elemental compositions of VR and hydrocracking samples (liquid and precipitate) were analyzed with an elemental analysis instrument (EA, Thermo Scientific Flash 2000 with thermal conductivity detector(TCD)) and an inductively coupled plasma atomic emission spectrometry (ICP-AES, Thermo Fischer Scientific iCAP 6500Duo). The compositions of macromolecules, which are saturates, aromatics, resins and asphaltenes (SARA), were analyzed by a SARA analyzer (Latroscan MK6s). The compositions of gas samples were quantified with gas chromatography (Supelco 80/100 Porapak Q column with TCD and HP-Plot Al_2_O_3_ KCl (Length = 50 m, Film thickness = 15 μm) capillary column with a flame ionization detector) calibrated with Agilent refinery gas analyzer mixture. The yield of coke was evaluated by a solvent extraction of hydrocarbon oils from a precipitate product. 0.5 g of precipitate product was mixed with excessive amount (about 100 ml) of toluene and wet solid particles were collected by filtration (with Macherey-Nagel 0.45 μm PTFE membrane filter). After complete drying of solvent, the final solid dry powder was weighed for the coke quantification. The yields for liquid fuel product were quantified by GC-Simdis (AC HT750 system, ASTM D7169) analysis for liquid and precipitate samples (only toluene soluble part).

## Additional Information

**How to cite this article**: Park, C. *et al*. Synthesis of Mesoporous a-Fe_2_O_3_ Nanoparticles by Non-ionic Soft Template and Their Applications to Heavy Oil Upgrading. *Sci. Rep.* **6**, 39136; doi: 10.1038/srep39136 (2016).

**Publisher's note:** Springer Nature remains neutral with regard to jurisdictional claims in published maps and institutional affiliations.

## Supplementary Material

Supplementary Information

## Figures and Tables

**Table 1:** The BET surface areas, BJH average pore sizes, and cumulative (between 1.7 nm and 350 nm in diameter) pore volumes for bulk and synthesized mesoporous hematite by different pH solutions

pH in starting solution	Morphology of primary crystal phase	BET surface area [m^2^ g^−1^]	Pore size [nm]	Pore volume [cm^3^ g^−1^]
–	Bulk	5.0	13.8	0.02
5	Oval	14.8	47.0	0.16
6	Oval	15.9	42.4	0.15
7	Mixed	30.9	18.8	0.16
8	Worm-like	42.6	19.2	0.18
9	Worm-like	44.4	20.9	0.22
