# Supplementary material for: Synthesis of Mesoporous α-Fe2O3 Nanoparticles by Non-ionic Soft Template and Their Applications to Heavy Oil Upgrading
Source: Sci Rep. 2016 Dec 14;6:39136. doi: 10.1038/srep39136 (PMC5155432; doi:10.1038/srep39136)
Supplement: Supplementary Information [file srep39136-s1.pdf]

Supplementary Information

Synthesis of Mesoporous α-Fe_2O_3 Nanoparticles by Non-ionic Soft Template and Their Applications to Heavy Oil Upgrading

Chulwoo Park[1,2], Jinhwan Jung[1], Chul Wee Lee[1,3], and Joungmo Cho[1,3*]

1. Research Center for Convergent Chemical Process, Korea Research Institute of Chemical Technology, Daejeon 34114, Republic of Korea

2. Kyoungbook National University, Daegu 41566, Republic of Korea

3. Department of Green Chemistry & Environmental Biotechnology, University of Science and Technology (UST), Daejeon 34113, Republic of Korea

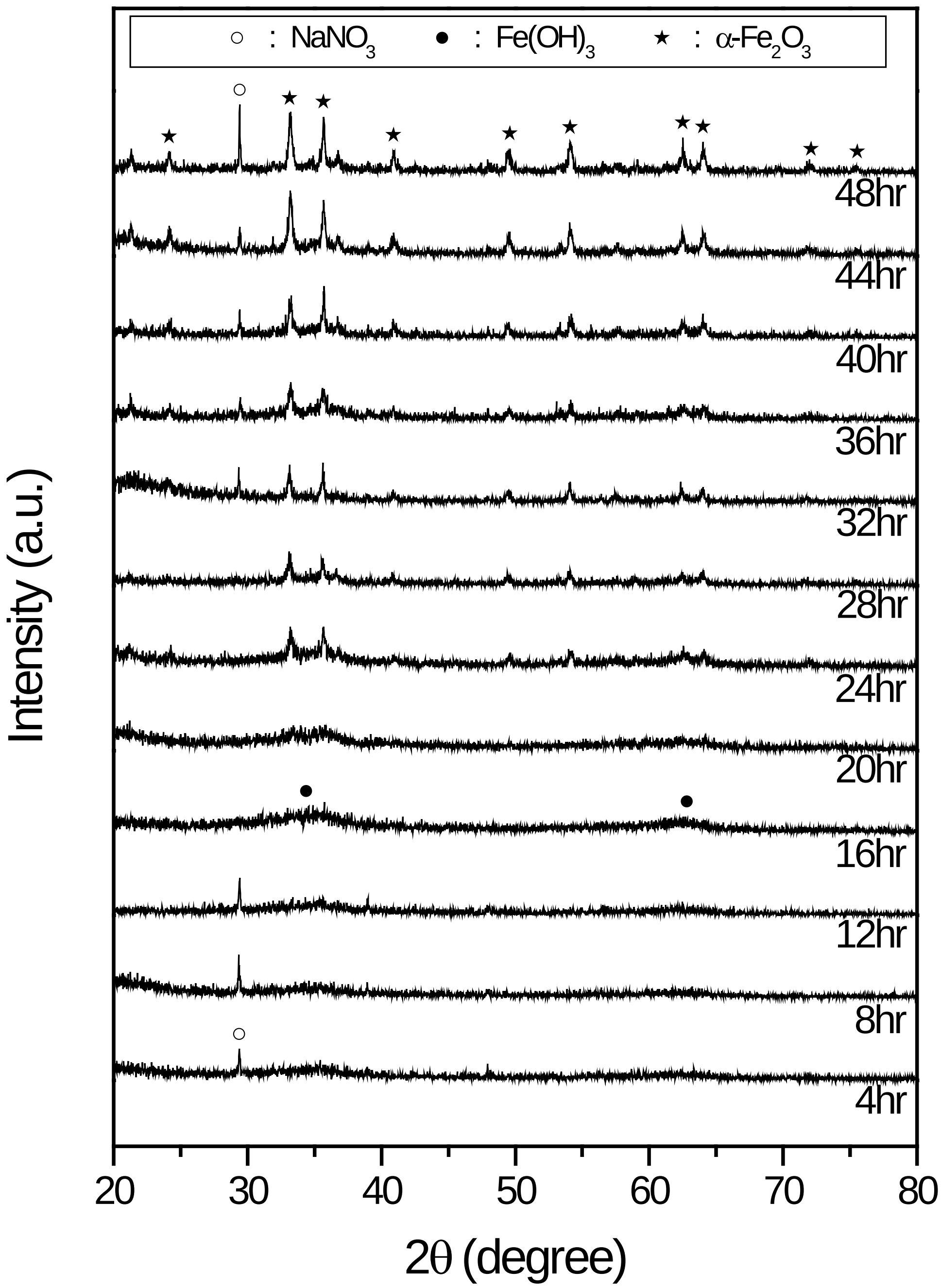

Figure S1 Measured XRD patterns to observe the formation of iron oxide crystal and the evolution of crystal phases.

The XRD analyses were carried out to mechanistically observe the formation of iron oxide crystals and phase transition over the time period of ripening. For this purpose, multiple samples were prepared with an aqueous solution of $Fe(NO_3)_3 \cdot 9H_2O$ (0.65wt%) initially adjusted to pH 7 by 10M NaOH solution and placed in an oven where the temperature was kept at 100°C. One of the samples was taken by every 4h and the solid

aggregate was prepared for XRD analysis after the filtration and drying. In the analysis, the samples were used without removal of salts to preserve pristine crystal phases. Fig. S1 shows the evolution of XRD patterns for the samples recovered over the ripening time of starting solution at a pH7. The initially formed solid phase mainly composed of ferrihydrite. The phase transition from ferrihydrite to hematite started to occur at around 24h for a given condition. Based on the ripening hours observed, the appropriate ripening time was decided to control the morphology and crystal phase in other experiments.

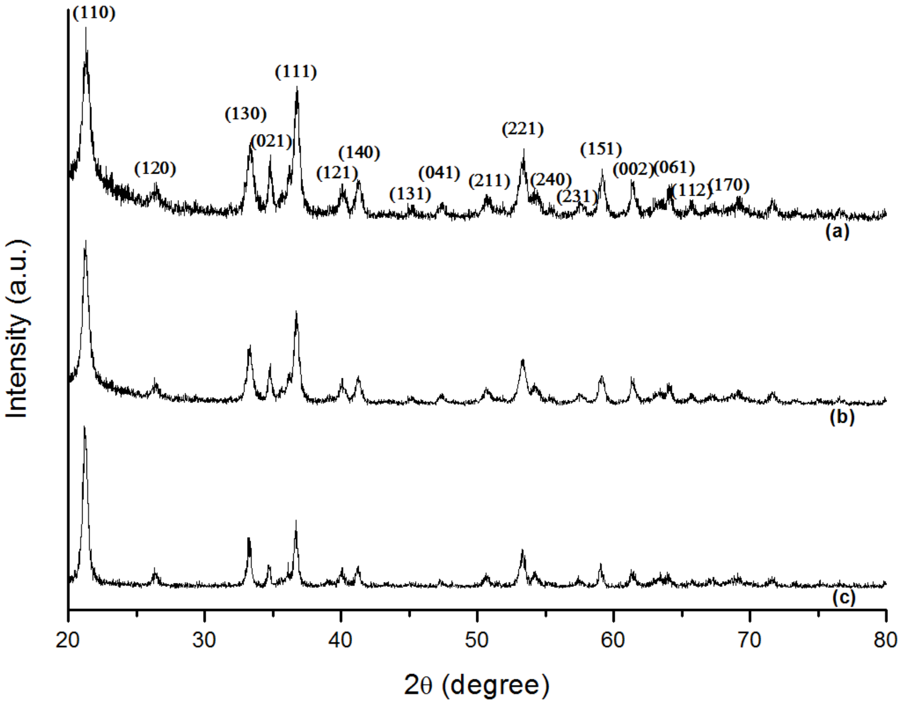

Figure S2. XRD patterns of as-synthesized iron oxide samples that are prepared at high pH conditions. The starting solutions are adjusted to (a) 10, (b) 11, and (c) 12, respectively.

Fig. S2 shows the XRD patterns for crystal structures synthesized at strongly basic conditions (pH 10-12). At these conditions, the primary crystal phases were obtained as a goethite. No significant difference of characteristic peaks for synthesized iron oxide was found once pH level of starting solution is beyond 9. The synthetic route at a strong basic condition basically didn't follow a pathway to produce α-Fe_2O_3, instead the

polymerization of iron oxyhydroxide formed the yellowish goethite precipitation.

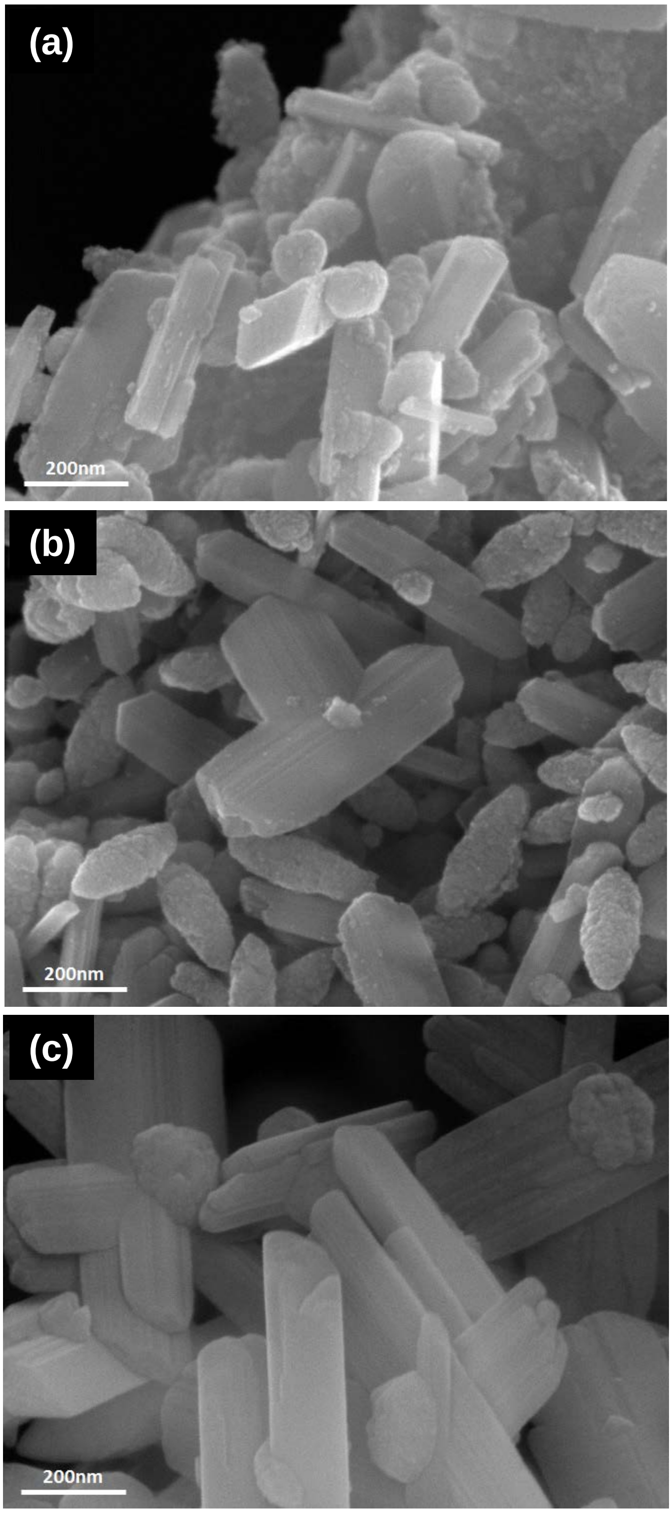

Figure S3. SEM images of as-synthesized iron oxide samples that are prepared at high pH conditions. The starting solutions are adjusted to (a) 10, (b) 11, and (c) 12, respectively.

Fig. S3 presents corresponding SEM photos of goethite synthesized at high pH conditions in the presence of P123. It has been well established in several literatures that the artificial synthesis of goethite at such conditions results in the growth of one dimensional structure, i.e. α-FeOOH nanorods. Although the same amount of P123 (with the case of worm-like hematite synthesis) was used in this case, no significant structure-directing effect of P123 was observed. Calcination of the samples (above 350°C) was performed in the presence of oxygen and clear transformation of the crystal phase to hematite could be observed in the XRD patterns. But we were not able to identify any unique mesoporous structures other than the similar morphologies shown in Fig. S3.

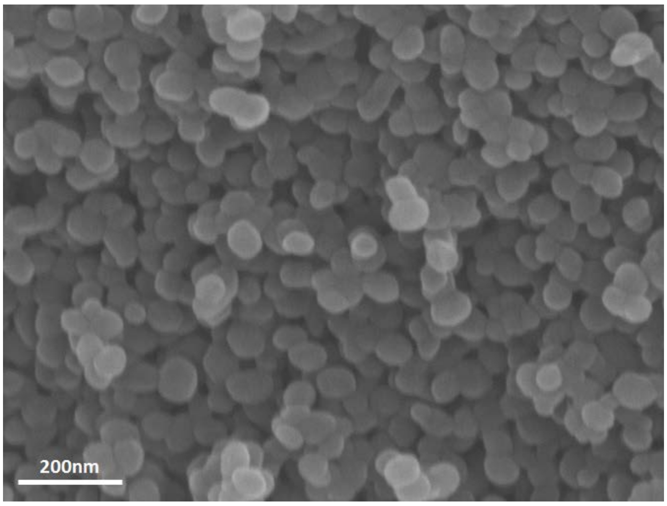

Figure S4. SEM image of calcined iron oxide nanoparticles prepared without hydrochloric acid.

Fig. S4 shows a SEM image for calcined iron oxide that was prepared from the solution at the same condition with the one described in the experimental part except but no addition of hydrochloric acid. The synthesis without hydrochloric acid led to mainly spherical iron oxide crystals even though we adjusted initial precursor solution to the same pH level. We were not able to clearly verify the role of HCl yet, but it seems help the formation of initial micellar structure by P123.

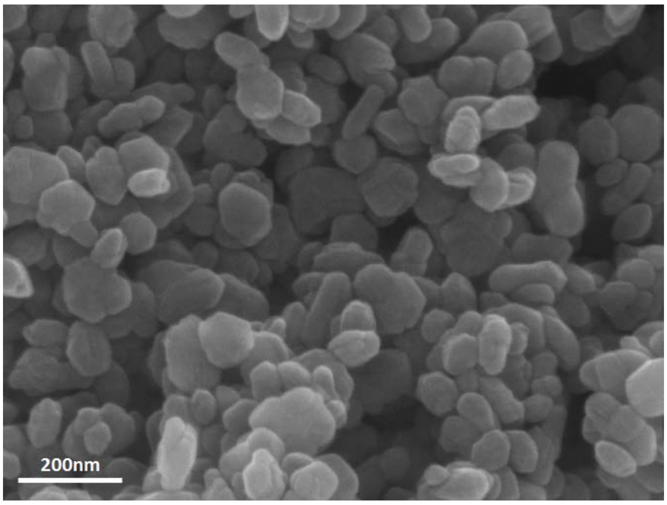

Figure S5. SEM image of calcined iron oxide nanoparticles prepared without P123.

Next, we checked the morphology of iron oxide synthesized without P123. In a similar way to previous, the aqueous solution was prepared by the same procedure nothing but zero concentration of P123. Basically, P123 is non-ionic. And thus it is expected that the net electronic charge of solution are not significantly influenced. In this case, any unique shapes or consistently mesoporous structures were not observed in the final iron oxide nanocrystals. The result implies that non-ionic P123 is necessary to derive the mesoporous iron oxide structure.

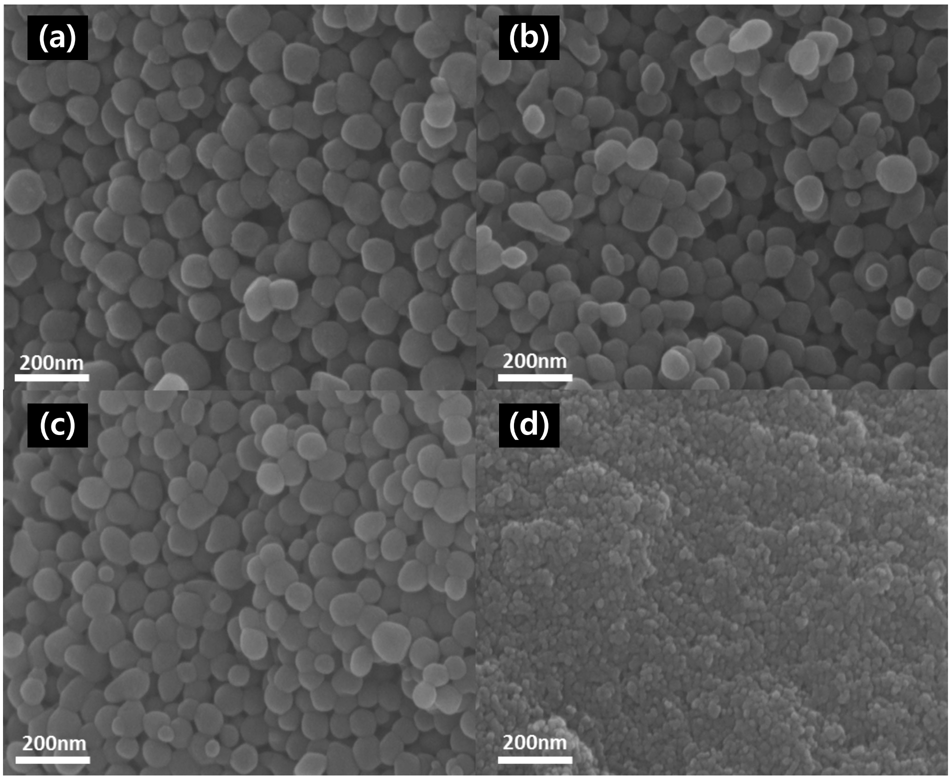

Figure S6. SEM image of calcined iron oxide nanoparticles prepared within different weight ratio of added precursor to surfactant. (a) [$Fe(NO_3)_3{\cdot}9H_2O$] : [P123] = 2:2, (b) 2:4, (c) 2:6, and (d) 2:10.

Finally, the morphologies of final iron oxides were observed when the concentrations of precursor and surfactant were varied (All other variables were set fixed). Fig. S6 shows the SEM images for the obtained iron oxide crystals. As described in the manuscript, the successful synthesis of worm-like iron oxide structure can be achieved only when the concentration ratio of [$Fe(NO_3)_3{\cdot}9H_2O$] : [P123] = 2:8. For the synthesis from different concentration ratios, the sphere-like morphologies were finally observed. For higher concentration ratios(Fig. S6 (a) through (C)), no significant size changes in primary particles were observed while high population of smaller particles less than 10nm were predominantly obtained for the case of [$Fe(NO_3)_3{\cdot}9H_2O$] : [P123] = 2:10. The small particle size and its discontinuity through the change of concentration ratio might be mainly caused by the spatial confinement with high concentration of surfactant rather than by micellar templates structured in the starting solution.

Table S1. The physical and chemical characteristics of vacuum residue used as a feedstock for the hydrocracking tests in this study.

Items	Used methods	Unit	Value
API Gravity	ASTM D4052-11		4.1
SG at 15.5°C	ASTM D5002-99		1.0303
Kinematic Viscosity (110°C)	ASTM D445-15a	cSt	1,030
Dynamic Viscosity (70-100°C)	ASTM D7042	cP	3,200-30,800
Carbon Residue	ASTM D4530-15	wt.%	23.26
SARA Analysis	ASTM D4124-09, ASTM D2007-03		
Saturates		wt.%	5.4
Aromatics		wt.%	60.5
Resins		wt.%	12.1
Asphaltenes		wt.%	22
Sulfur	ASTM D4294-03	wt.%	5.5
Nickel		wppm	74
Vanadium		wppm	325
Fuel compositions	ASTM D7169		
Naphtha (IBP-177°C)		wt.%	0.0
Middle Distillate (177-343°C)		wt.%	0.0
Gas Oil (343-524°C)		wt.%	10.1
Residue (524°C+)		wt.%	89.9

In the commercial slurry-phase hydrocracking processes employing iron oxide catalysts, the catalysts are applied as a once-through mode mainly because the catalyst recycle adds more complexity and subsequent high investment cost which could not be offset by the reduction in operating cost. In these technologies, the price for catalysts occupies very small fraction among other operating costs. Nevertheless, the recycle tests of worm-like may have a significance to investigate stability of the catalyst, in case expensive metals, such as molybdenum, are added although the amount used is relatively small. In this respect, we have tested the catalytic activities with the recycled catalysts by observing crystal structure changes with XRD and SEM images. The properties of feedstock used for hydrocracking tests in the current study are listed in Table S1. The corresponding results (Fig. S7 through Fig. S10) are described in the followings

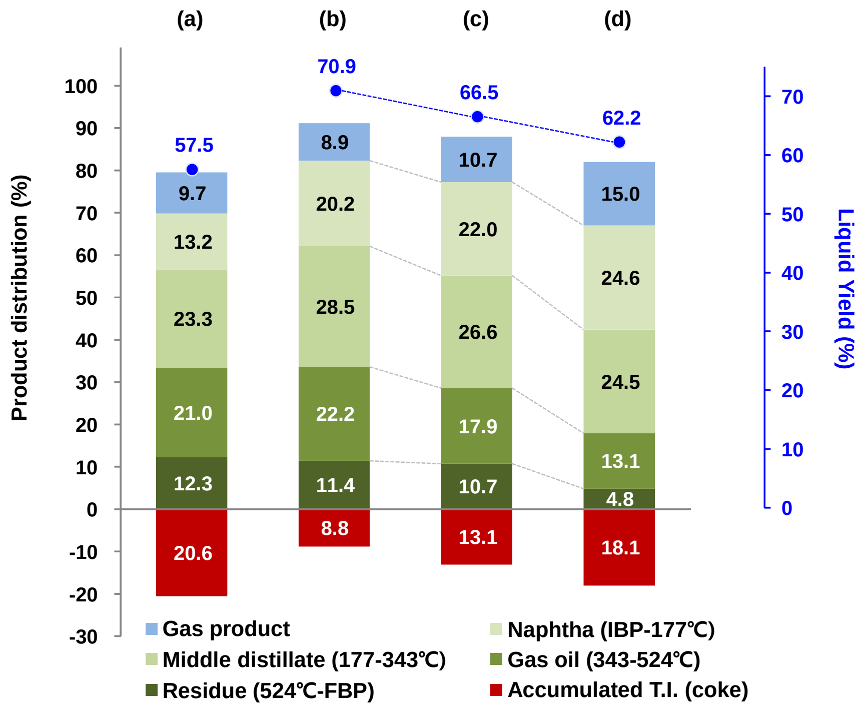

Figure S7. The product distributions after the hydrocracking of (a) no catalyst addition, (b) the first run with 2%Mo/α-Fe_2O_3(pH9), (c) the second run, and (d) the third run by recycling the catalysts and solid cokes. The catalysts recycle tests are performed by making up the same amount of new feedstock. Accordingly, the amount of cokes in second and third runs indicates the accumulation of toluene insoluble as known the reaction route to coke formation is irreversible. All other reaction conditions are fixed to the same with the first hydrocracking reaction.

The catalytic activity and stability changes were investigated by recycling the catalysts with heavy oils make-up. In each run, the catalyst was provided without air regeneration or separation from the solid cokes to preserve active form of the iron-based catalyst. In slurry-phase hydrocracking of heavy residual hydrocarbons, iron oxide catalysts are converted into the sulfide form, i.e. pyrrhotite ($Fe_{(x-1)}S_x$; JCPDS 29-0723), which is an active form for the hydrogenation of hydrocarbons. The structural changes of worm-like iron oxide are further discussed later. As shown in Fig. S7, for a given number of recycle tests, the catalysts shows a stable hydrocracking abilities including high conversion and high yield of valuable products (the yield of naphtha and middle distillate is kept at around 49%) while there are some variations in the amount of coke and gas formation.

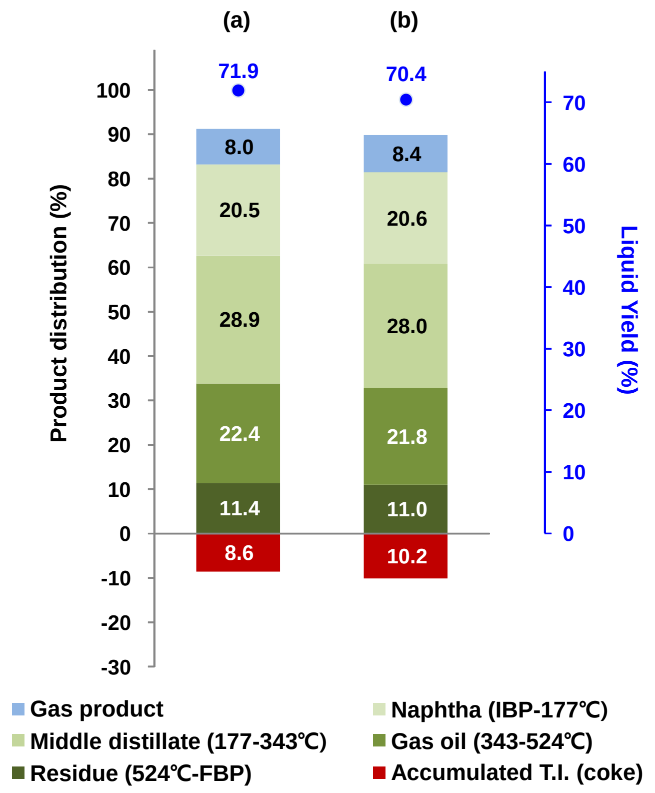

Figure S8. The product distributions after the hydrocracking with (a) the catalyst in the present work and (b) the iron oxide based commercial catalyst.

Fig. S8 shows the compared catalytic performances for the slurry-phase hydrocracking of heavy residual hydrocarbon over the synthesize catalysts in the current study and the commercially available catalysts (RM Scarlet SPN grade provided by KC Co.; Average particle size ~13μm). Both catalysts shows similar liquid product yield distributions, but the worm-like iron oxide catalysts shows a better performance in terms of overall liquid product yield, conversion, and less amount of gas and coke formations.

The XRD patterns of recovered iron-based catalysts after the hydrocracking tests (presented in Fig. S7 and Fig. S8) are depicted in Fig. S9. The initial hematite crystalline structures are converted into the pyrrhotite forms for both the catalysts synthesized in the current study and the commercial iron oxide catalysts. The characteristic peaks of pyrrhotite disappear along the repeated hydrocracking tests for the recycled catalysts.

This ascribed to the relatively high amount of coke contents in the sample burying the characteristic peaks of the active metal forms.

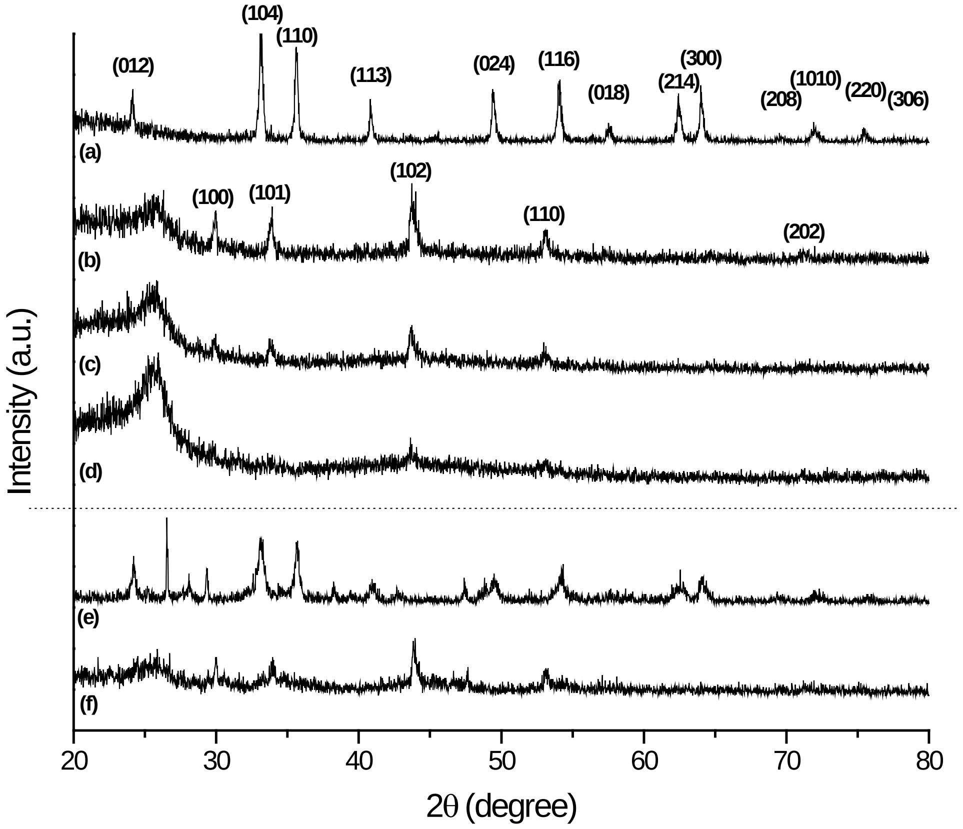

Figure S9. XRD patterns for (a) fresh 2%Mo/α-Fe_2O_3(pH9), the collected solid mixture of coke and catalyst after the hydrocracking reactions with (b) the first run, (c) the second run, (d) the third run, and for (e) the commercial iron oxide catalyst and (f) the recovered solid mixture after the hydrocracking reaction.

Fig. S10 shows the SEM images for the morphologies of pyrrhotite forms after the hydrocracking tests. The samples (originally started with oval and worm-like shape hematite nanostructures) are prepared by repeated toluene washing and filtration of solid mixture collected after the hydrocracking tests. It is notable that the morphologies of original worm-like hematite were not significantly destroyed while there was a major crystalline phase transformation of hematite into pyrrhotite even for a single hydrocracking run (as observed in XRD patterns in Fig. S9).

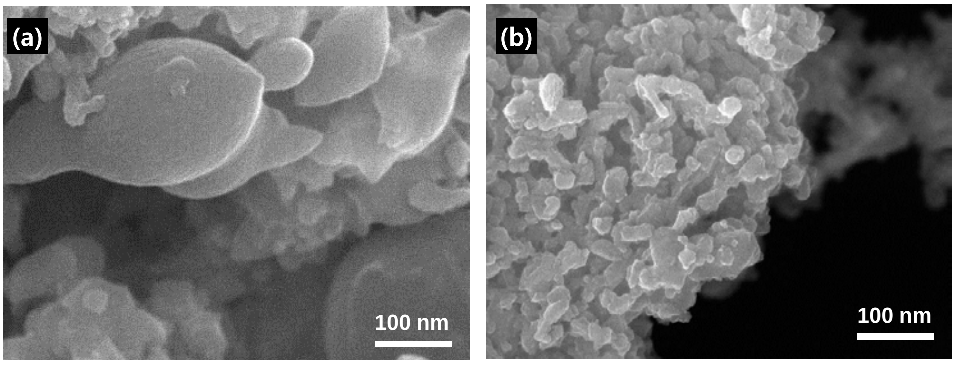

Figure S10. SEM images for pyrrhotite nanostructures obtained after the hydrocracking reactions started with (a) oval and (b) worm-like shape hematite nanostructures.
